# Extracellular electron transfer genes expressed by candidate flocking bacteria in cable bacteria sediment

**DOI:** 10.1128/msystems.01259-24

**Published:** 2024-12-19

**Authors:** Jamie J. M. Lustermans, Mantas Sereika, Laurine D. W. Burdorf, Mads Albertsen, Andreas Schramm, Ian P. G. Marshall

**Affiliations:** 1 Center for Electromicrobiology, Department of Biology, Aarhus University, Aarhus, Denmark; 2 Microbial Systems Technology Excellence Centre, University of Antwerp, Wilrijk, Belgium; 3 Department of Biology, Research Group Geobiology, University of Antwerp, Wilrijk, Belgium; 4 Center for Microbial Communities, Aalborg University, Aalborg, Denmark; University of Illinois at Chicago, Chicago, Illinois, USA

**Keywords:** cable bacteria, flocking bacteria, extracellular electron transfer, interspecies electron transfer, sediment

## Abstract

Cable bacteria, filamentous sulfide oxidizers that live in sulfidic sediments, are at times associated with large flocks of swimming bacteria. It has been proposed that these flocks of bacteria transport electrons extracellularly to cable bacteria via an electron shuttle intermediate, but the identity and activity of these bacteria in freshwater sediment remain mostly uninvestigated. Here, we elucidate the electron exchange capabilities of the bacterial community by coupling metagenomics and metatranscriptomics to 16S rRNA amplicon-based correlations with cable bacteria over 155 days. We identified candidate flocking bacteria as bacteria containing genes for motility and extracellular electron transfer including synthesis genes for potential extracellular electron shuttles: phenazines and flavins. Based on these criteria, 22 MAGs were from candidate flockers, which constituted 21.4% of all 103 MAGs. Of the candidate flocking bacteria, 42.1% expressed extracellular electron transfer genes. The proposed flockers belonged to a large variety of metabolically versatile taxonomic groups: 18 genera spread across nine phyla. Our data suggest that cable bacteria in freshwater sediments engage in electric relationships with diverse exoelectrogenic microbes. This community, found in deeper anoxic sediment layers, is involved in sulfur, carbon, and metal (in particular Fe) cycling and indirectly utilizes oxygen here by extracellularly transferring electrons to cable bacteria.

**IMPORTANCE:**

Cable bacteria are ubiquitous, filamentous bacteria that couple sulfide oxidation to the reduction of oxygen at up to centimeter distances in sediment. Cable bacterial impact extends beyond sulfide oxidation via interactions with other bacteria that flock around cable bacteria and use them as electron acceptor “shortcut” to oxygen. The exact nature of this interspecies electric interaction remained unknown. With metagenomics and metatranscriptomics, we determined what extracellular electron transport processes co-occur with cable bacteria, demonstrating the identity and metabolic capabilities of these potential flockers. In sediments, microbial activities are sharply divided into anaerobic and aerobic processes, with oxygen reaching only millimeters deep. Cable bacteria extend the influence of oxygen to several centimeters, revealing a new class of anaerobic microbial metabolism with cable bacteria as electron acceptors. This fundamentally changes our understanding of sediment microbial ecology with wide-reaching consequences for sulfur, metal (in particular Fe), and carbon cycling in freshwater and marine sediments.

## INTRODUCTION

Cable bacteria are filamentous sulfide oxidizers, that can become centimeters long, and modify the environment when they are metabolically active (1–3). The cable bacteria respire oxygen in the oxic zone of sediments they live in, while oxidizing sulfide in sulfidic parts to conserve energy (2). This results in physical separation of the oxidative and reductive metabolic processes, which is achieved by transport of electrons from sulfide oxidation through their filaments upwards to where the oxygen reduction takes place, making them an electric conduit (4).

Cable bacteria belong to the genera *Candidatus* Electronema and *Candidatus* Electrothrix, which are part of the *Desulfobulbaceae* (5). Thus far, cable bacteria have not been isolated in pure culture and remain in sediment enrichments or natural microbial communities (6). In both natural settings (lakes, estuaries, coastal, and deep sea) (2, 5, 7–10) and sediment enrichments (6), cable bacteria impact microbial community composition and activity. This has been observed in several instances such as chemoautotrophic sulfide-oxidizing *Gamma*- and *Epsilonproteobacteria* (*Campylobacterota*) that accrued more carbon around active cable bacteria, iron reducers that appeared in higher abundance in presence of cable bacteria, or increased sulfate concentrations that stimulated sulfate reduction when cable bacteria were metabolically active (7–11). Some associations likely arose from the geochemical changes (8, 9). However, not all associations could be explained by these, which led to suggestions that exoelectrogenic interactions may occur with cable bacteria (11–13).

An example of potential exoelectrogenic interactions with cable bacteria is the flocking behavior that occurs around *Ca*. Electronema aureum GS in freshwater sediment (12, 14). On microscope slides, cable bacteria were discovered to have hundreds of bacteria swimming actively around individual cable bacteria filaments, seemingly without making direct physical contact with the cable bacteria (12). Flocking appears consistently with active, electron-conducting cable bacteria, and the morphology of flocking bacteria changes over time, suggesting different bacteria flock at different time points (12, 14). Bjerg and colleagues (12) suggested that flocking bacteria exhibit a form of extracellular electron transfer (EET) that may be electron shuttle mediated. Electron shuttles are compounds that can be oxidized and reduced without being consumed and can be used by microbes to respire insoluble electron acceptors such as metal particles or electrodes (15, 16). Bjerg and colleagues (12) hypothesize that the flockers would donate electrons to such a shuttle, followed by the cable bacteria coupling the oxidation of these shuttles to the reduction of O_2_ in the oxic zone at several millimeters distance from the flocking. This would explain the occurrence of, for example, aerobic bacteria capable of EET, in an anoxic environment donating electrons to cable bacteria instead of to a terminal electron acceptor. EET could be achieved by using commonly synthesized electron shuttles such as flavins and phenazines, or humic substances which are found in organics-rich sediments (12, 17, 18). However, the potential EET mechanism behind this flocking behavior remains unknown.

Several pathways used for EET are known from model organisms: *Shewanella oneidensis* MR-1, *Geobacter sulfurreducens*, *Pseudomonas putida*, *Thermincola potens* JR, *Rhodopseudomonas palustris* TIE1, *Enterococcus faecalis*, and *Sideroxydans lithotrophicus* ES-1, which is thought to oxidize iron via EET (15, 19, 20). Ten porin-cytochrome complexes (pccs) (2 Mtr’s, Pio, Mto, Cyc2, TherJR_2595, OmabcB, and its three homologs) that export electrons and oxidize substances outside of the cell have been described (19, 21). EET appears widespread, making it likely that other microorganisms have different pathways to perform EET (21–24). Shuttle-based EET requires redox-active compounds that could be excreted by microorganisms if not already in the environment (17, 25, 26). Calculations made by Bjerg and colleagues (12) showed that low (nanomolar) concentrations of shuttles in the sediment could fuel the potential electric interaction that the hundreds of flockers were suggested to perform. For shuttle synthesis and excretion of the most commonly used shuttles (flavins and phenazines), specialized proteins are used such as PhzABCDEF, IpdG, and MexGHI-OpmD for phenazines, and Bfe, YeeO, and RibBA/X for flavins (27–31).

With this knowledge, a microbial community’s potential for EET may be determined through presence and expression of EET-encoding genes using metagenomics and metatranscriptomics. We combined this with previously and newly published 16S rRNA amplicon data collected over 76–155 days (14) to determine whether the microbial community in a cable bacteria enrichment has EET potential. We determined specific taxonomic groups in this complexity-reduced community, which correlate with *Ca*. Electronema and looked at their genetic potential and usage of EET.

## MATERIALS AND METHODS

The samples originated from three time series experiments with sediment from two different lakes. The first experiment (TS1) was a 76-day incubation of *Candidatus* Electronema aureum GS (6) in autoclaved Vennelyst Park (Denmark) sediment. The second was a 155-day incubation (TS2) under the same conditions as TS1. For the last experiment (BS1), sieved Brabrand Lake sediment was incubated for 76 days. The *Ca*. E. aureum GS enrichment has been maintained as a laboratory enrichment (6), reducing its community complexity since inoculation of autoclaved sediment occurred by transfer of a cable bacteria-containing sediment clump (~0.5 g–2 g). In contrast to the GS enrichment, the Brabrand Lake sediment was collected specifically for this study for its “natural” community to compare the GS sediment with a more natural environment.

16S rRNA sequences derived from the time series with *Ca*. Electronema aureum GS were used from Lustermans et al. (14) with exception of the 155-day time point, which was added for this study, but collected and generated similarly as described by Lustermans et al. (14).

### Sediment collection and microsensor set-up

Black (typically indicating the presence of sulfide) freshwater sediment was retrieved from Brabrand Lake, Denmark (56.140458, 10.144352) at a water depth of 3 m–4 m. The sediment was stored with overlying water at 15°C for 2 weeks to ensure anoxia which removes macrofauna.

Before incubation, sediment was homogenized, sieved (pore size: 0.5 mm), and distributed into core liners that were closed with a rubber stopper at the bottom. Settling occurred after 24 h, after which the sediment surface was aligned with the core liner edge and cores were then submerged in autoclaved tap water (Fig. S1). The aquarium was kept at 15°C, covered with aluminum foil to prevent algae formation, equipped with air circulators and a lid to prevent excessive evaporation. Overlying water was replenished and refreshed multiple times during incubation.

Microsensor measurements for O_2_ and EP (electric potential), to determine the active current-generating (sulfide oxidation) zone, were performed and analyzed as described in Lustermans et al. (14). All sediment samples were collected sacrificially by cutting cores based on geochemical zonation, as previously described (14).

### 16S rRNA amplicon community analysis

The taxonomically classified collection of V3-V4 16S rRNA amplicons were generated after total RNA extraction in a previous study (14) or produced in this study using the same procedure. Correlation analysis was carried out on (i) the complete set (TS1: nine time points, 16 samples, TS2: 10 time points, 20 samples) of *Ca*. Electronema aureum GS samples, (ii) all natural samples (BS1, Brabrand Lake, *Ca*. Electronema sp.: nine time points, 11 samples), and (iii) subsets of the cable bacterial growth phase to avoid spurious correlations resulting from increased relative abundance of other 16S rRNA sequences relative to cable bacteria. Growth was defined as increase of cable bacterial relative abundance based on 16S rRNA sequencing: days 3–33 for the first set of amplicons (TS1) and days 2–27 for amplicons from the second time series (TS2) (14). Fractional abundances of ASVs (amplicon sequence variants) were calculated, followed by pruning of ASVs unclassified to genus level. The remaining classified ASVs were then agglomerated into genera with Phyloseq v.1.42.0 (32). Only genera with a fractional abundance above 0.09% in more than three samples were used for correlation calculations. Spearman correlations were performed at genus level against *Ca*. Electronema (sp.), followed by a *P*-value adjustment to account for multiple testing (R v.4.2.2; cor.test [method = Spearman), *P*_adj_). Heatmaps were generated using ampvis2 v.2.6.4 (33).

### Metatranscriptomics

Metatranscriptomes were prepared of three time points (TS1: days 3, 26, 33) (14). Samples represent low and high cable bacteria activity/abundance for comparison. Total RNA was extracted in duplicate from the active current-generating zone (anoxic bulk sediment) using the RNeasy PowerSoil Total RNA kit (Qiagen) and concentrated with the kit RNA Clean & Concentrator (Zymo Research, USA) including an in-column DNase treatment (Ambion, USA). Metatranscriptome sequencing (2 × 50 bp, 50 mio reads per sample) was done with Illumina HiSeq (Illumina, USA) without prior rRNA removal (DNASense, Denmark). The sequences were trimmed, and size filtered using Trimmomatic v.0.39 (34).

### Metagenomics

Two metagenomes derived from the *Ca*. Electronema aureum GS enrichment were used for mapping metatrancriptomic data: a short-read Illumina data set from a trench-slide incubation where flocking was observed (12), and a long-read Oxford Nanopore metagenome from anoxic bulk sediment (35). A trench slide consists of two glued-together microscopy slides, with a central cavity in the upper one. This is filled with sediment, covered with anoxic tap water and a cover slip (12). Cable bacteria glide from the central sediment compartment (which also releases sulfide) onto an observable plane toward inward-diffusing oxygen.

Preprocessing, sequencing, and binning of the metagenomes were carried out as described previously (12, 35), with the resultant metagenome-assembled genomes (MAGs) incorporated into this study.

FastANI v.1.32 (36) was run against MAGs from both sources to remove duplicates. If two near-identical (>98.5% average nucleotide identity [ANI]) MAGS were found, the most complete MAG was chosen for further analysis as calculated by CheckM2 v.1.0.1 (37). Genes of each MAG were identified and annotated using PROKKA (38), followed by taxonomic classification of the bins with GTDB-tk v.2.1.0 and database r207 (39). The final bin collection was run through KOfamscan v.1.3.0 (40) with a 1E−6 cutoff value and compared with Blastp (41) with a 1E−6 cutoff value to an inhouse-prepared database containing EET proteins from various origins (Table S1).

### Mapping and *de novo* analyses

Trimmed and filtered sequences were mapped against the final MAGs with bbmap v.39.01 (42). This was then used for differential gene expression analysis with DESeq2 v.1.39.8 (43) (R v.4.2.2). The expressed genes were compared to the previously mentioned Blastp analysis of the metagenomes and results were compiled (Tables S1 to S3).

*De novo* assembly was done on the pooled metatranscriptome sequences using rna-SPAdes v.3.15.4 (44). Protein-coding genes were identified in assembled transcripts using FragGeneScan v.1.30 (45). Genes were annotated and identified as EET genes as described for the metagenomes. Taxonomy of genes was determined with eggNOG-mapper v.2.0.1 (46). Discovered EET genes were compared to the metagenome and correlating taxa.

## RESULTS

### 16S rRNA amplicon analyses

We acquired 36 samples from the *Ca*. Electronema aureum GS time series. These originated from two time series: TS1 (76 days, nine time points) and TS2 (155 days, 10 time points). From the Brabrand Lake sediment time series (BS1), we generated 11 samples over 76 days (nine time points). All samples had more than 3,909 reads with an upper limit of 132,888 reads and a median of 22,202.

Positive correlations (*P*_adj_ <0.05, ρ ≥ 0.6) were determined between rRNA of the genus *Ca*. Electronema and other genera in TS1 and TS2 and in the natural sediment BS1, which contained several strains of *Ca*. Electronema.

**Fig 1:**
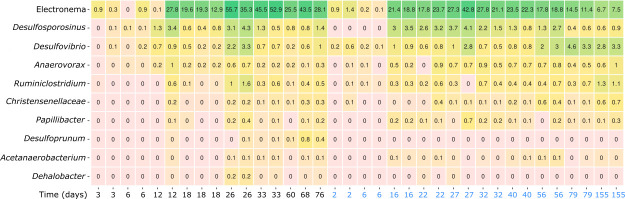
Heatmap of both time series containing *Ca*. Electronema aureum GS enriched sediment showing the genera that correlated positively with *Ca*. Electronema aureum GS over time (2–155 days, black TS1, blue TS2). Numbers on heatmap show percentage abundances.

In the inoculated enrichment *Ca*. Electronema aureum GS, rRNA increased in relative abundance to ~55% of the community on days 26–33, after which it decreased slowly to day 155 without disappearing (Fig. 1). The genera *Desulfosporosinus*, *Dehalobacter*, *Papillibacter*, *Acetanaerobacterium*, and *Desulfoprunum* positively correlated with *Ca*. Electronema aureum throughout the enrichment. *Christensenellaceae* R-7 group, *Desulfovibrio*, *Papillibacter*, *Anaerovorax*, and *Ruminiclostridium* correlated positively during the cable bacteria’s growth phase (days 3–33).

Different correlations were found when the two time series were separated into TS1 and TS2 (SI Fig. 1 and 2). In TS1, *Dehalobacter*, *Ruminiclostridium*, *Christensenellaceae* R-7 group, *Desulfurispora,* and *Acetanaerobacterium* correlated positively with cable bacteria during the 76 days of incubation. In TS2, *Desulfosporosinus* was the sole positive correlation with cable bacteria over 155 days.

**Fig 2:**
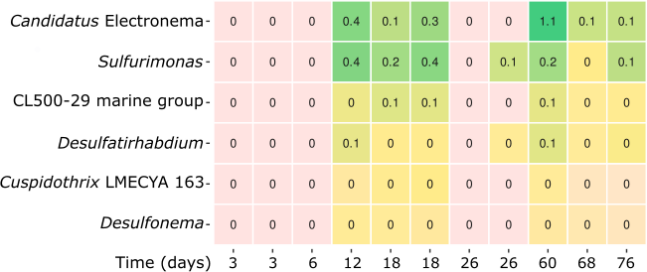
Heatmap of naturally occurring *Ca*. Electronema sp. in Brabrand Lake, showing the genus-level taxa that correlated positively with cable bacterial succession over time (3–76 days). Numbers on heatmap show percentage abundances.

In the natural sediment BS1, the cable bacteria, *Ca*. Electronema sp., rRNA showed lower relative abundance than in the two enrichments with a maximum of 1.1% on day 64 (Fig. 2). The positive correlations that were found in the natural cable bacteria cores during 76 days were with: *Desulfonema*, *Sulfurimonas*, *Desulfatirhabdium*, CL500-29 marine group (*Ilumatobacteraceae*), and *Cuspidothrix* LMEYCA_163.

### Metagenomics and metatranscriptomics analyses

**Fig 3:**
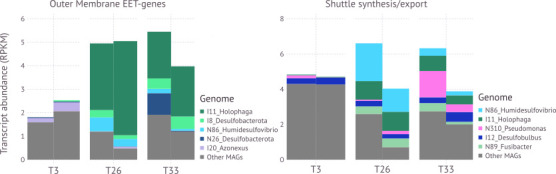
Fractional abundances (RPKM) of transcripts of pccs and electron shuttle genes were mapped against MAGs for low (day 3) or high (days 26,33) relative cable bacterial abundance. Only the five highest scoring MAGs were shown individually, other are clustered in “Other MAGs.” The pcc genes included *ndh3, eetB, dmkA, omabcB, omabcC, therJR_2595, therJR_0333, therJR_1122, cyc2, mtrABC, mtrDEF, pioAB, mtoAB, dmsAEF, cwcA, extEFG, extBCD, omcA, omcS, omcZ*. The shuttle genes used were: *yeeO, bfe, ribBA, ribBX, ribD, ribE1, ribE2, ipdG, phzDEFG, mexGHI, OpmD*.

We determined presence or absence of genes for motility, EET, or electron shuttle-related genes in the metagenomes derived from the *Ca*. Electronema aureum GS enrichment, and observed whether transcripts of these genes could be detected by RNA sequencing. The metagenomes were binned into 103 unique MAGs, of which 85 were of high quality (>90% complete and <5% contaminated). We identified 5,083 EET, shuttle synthesis, and excretion genes in the MAGs, to which 959 genes were mapped from the metatranscriptomes (Table S1). Only 164 of these overlapped with the *de novo*-assembled metatranscriptomes, which yielded another 534, unmapped EET-related gene fragments. To determine whether EET-related transcripts were more prevalent when there were more cable bacteria (days 26, 33) compared to when they were in lower abundance (day 3), significant differences in transcript abundance of outer membrane EET or electron shuttle genes were analyzed for the MAG-mapped transcripts (Fig. 3). The *de novo*-assembled transcript sequences were too fragmented to accurately quantify, but they indicate that the genes found in MAGs are not an exhaustive description of all EET activity in the community, as a large part of unmapped EET-related genes was discovered among the *de novo*-assembled metatranscriptome sequences. As expected, transcripts for 79% of *Ca*. Electronema aureum GS genes were significantly more abundant when the bacterium was in high abundance (Table S2). Significantly more abundant gene transcripts during high cable bacterial abundance (days 26, 33) versus low abundance (day 3) as a percentage of overall mapped gene transcripts for each MAG varied widely from less than 0.5% to 41% (Table S2). There were 22 out of the 103 MAGs that had no significantly more abundant transcripts compared between the cable bacteria abundances (Table S2).

### Porin cytochrome complexes and outer membrane cytochromes (omcs)

**Fig 4:**
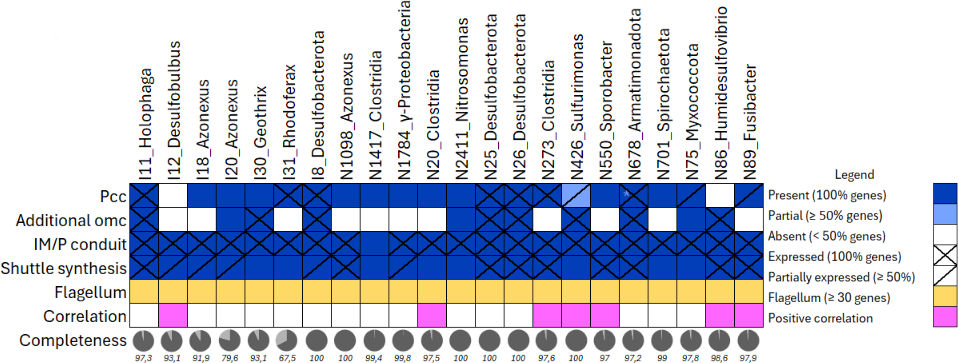
Selection of MAGs identified as potentially flocking around cable bacteria, with genetic potential and gene expression related to EET (blue); genetic potential related to flagellar motility (yellow); positive correlations based on 16S rRNA genus level (pink); and genome completeness (circles and percentages below). Pcc, porin cytochrome complex; omc, outer membrane cytochrome (including *pioA, cyc2, mtoA, cwcA, omcA,B,C,S,Z, therJR_2595, extC,D,F, mtrA,F*); IM/P, inner membrane/periplasmic.

Complete sets of pcc encoding genes, which are necessary for microbes to perform EET, were found in 18 bacterial genomes (Fig. 4). And many other MAGs contained incomplete versions of pccs (Table S1). A single bin contained four copies of the single-gene pcc *cwcA*, which spans the cell wall in the Gram-positive EET performer *Thermincola* sp.

In total, we found 22 potential EET-performing bacteria. Pcc-encoding genes (including *dmkA*, *ndh3, eetB,* and *cwcA, cyc2* as three complete pccs, excluding *mtoD* and *pioC* as these are not part of the membrane-bound part of the porin complex) were transcribed by 57 MAGs of the 103 total MAGs (Table S1). Fifty-four of these 57 MAGs transcribed at least one gene of a pcc on day 3, 26, or 33. I11_*Holophaga* transcribed the highest amount of outer membrane EET genes, and did so only during high cable bacteria abundance (days 26, 33; Fig. 3). The other top 5 transcribers of outer membrane EET genes, I8_*Desulfobacterota*, N86_*Humidesulfovibrio*, N26_*Desulfobacterota,* and I20_*Azonexus*, were found to transcribe their genes at low and high cable bacterial abundance (Fig. 3). However, with exception of I20_*Azonexus*, these MAGs all transcribed more outer membrane EET genes during high cable bacterial abundance. All *Clostridia* (a genus positively correlated with cable bacteria according to 16S rRNA data)-classified MAGs had *eetB* and two or more other *Enterococcus faecalis*-type pcc genes (Fig. 4; Table S1). The *Sulfurimonas* expressed most genes found in the *Shewanella oneidensis* pathway, *pioA*, and *mtoA* which are all parts of pccs that appear essential for EET (Fig. 4; Table S1).

Genes encoding cytochromes on the outside of the outer membrane (*omcA, omcS, omcZ*, and *therJR_2595*) were present in 22 MAGs and transcribed by 5 of these (Fig. 4; Table S1). *omcA* was found in only one MAG, while the other genes were identified in at least two different MAGs. One of the top 5 transcribing MAGs, I8_*Desulfobacterota* transcribed *therJR_2595, omcS,* and *omcZ* (Table S1). Omcs (including those from the pccs) were identified in 31 MAGs of which (multiple) transcripts were mapped to 15 MAGs (Table S1).

### Periplasmic (P) and inner membrane (IM) cytochromes

Aside from a pcc, microbes need a conduit to transport electrons from the cytoplasm to the outer membrane. Most MAGs encoded and, with few exceptions, transcribed *macA, cbcL, imcH, menB, menE,* and *fccA/3* genes for this purpose (Fig. 4; Table S1). While other conduits, *cymA, imdcA, pdcA, imoA,* were only sporadically transcribed and/or encoded (Table S1), IM/P conduits were transcribed by 78 out of the 103 MAGs (Table S1).

Significantly more of the EET genes (encoding pccs, omcs, P/IM cytochromes) that were transcribed during high cable abundance belonged to seven MAGs (I11, I18, I20, I8, N1098, N273, N89).

### Electron shuttles and motility

Electron shuttle secretion and synthesis genes were transcribed by 64 MAGs (80 including the MexGHI-OpmD pump, which is not exclusively used for excretion of electron shuttles). Flavin production based on the *ribBA/X* gene was transcribed by 27 bacteria and one archaeon. Exporters for flavins encoded by *bfe* or *yeeO* were transcribed by 12 different bacteria (Table S1). One of the phenazine synthesis genes, *phzE*, was transcribed by 25 bacteria, while 54 bacteria transcribed the *ipdG* phenazine regulator gene of *Pseudomonas aeruginosa* (Table S1). Both archaeal bins, identified as methanogens, had the potential to produce electron shuttles, while one also transcribed two genes for shuttle production (Table S1). Six of the bacteria that correlated positively with cable bacteria transcribed synthesis and excretion genes for flavins or phenazines (Fig. 4). The cable bacteria transcribed genes for flavin and phenazine production (Fig. 4; Table S1). Electron shuttle synthesis gene expression (including *mexGHI, OpmD*), additional to those mapped to the MAGs, was also discovered in the *de novo* assembly. N86_*Humidesulfovibrio* transcribed the highest amount of shuttle synthesis-related genes of all MAGs and did so mostly during high cable bacteria abundance (Fig. 3). The other top 5 transcribers of shuttle synthesis genes were I11_*Holophaga*, N310_*Pseudomonas*, I12_*Desulfobulbus*, and N89_*Fusibacter*. These all transcribed genes during low and high cable bacterial abundance, but transcribed more during higher cable bacterial abundance, with exception of N89_*Fusibacter* which only transcribed during high cable bacterial abundance (Fig. 3).

Out of the 103 MAGs, 66 bacteria were likely motile by flagella since they contained more than 30 of the 55 needed genes according to the KEGG database (Table S1).

## DISCUSSION

We collated the three different data sets (amplicons, metatranscriptomics, and metagenomics) to identify candidate flockers based on (i) motility, (ii) EET genes and transcription thereof, and (iii) 16S rRNA correlations or EET genes that were significantly more expressed during high cable bacteria abundance.

In this discussion, we will consider information on both marine and freshwater cable bacteria to evaluate the freshwater systems that we used, because most microbial ecology and geochemical research has been done on cable bacteria-containing marine sediments.

For item ii, we focused on complete pcc homologs to any of the model EET organisms (19–21, 47–49). We assumed that discovered hybrid pccs were functional if a complete outer membrane-crossing complex could be created, since Conley et al. (50) showed that *Vibrio natriegens* uses a hybrid EET pathway combining proteins from *Aeromonas* and *Shewanella*.

Flocking bacteria were exclusively found around actively electron conducting cable bacteria (12, 14). For this, cable bacteria would function as alternative electron acceptor through shuttle-based EET to calculated concentrations of 2 nM–187 nM electron shuttle (12). Thus, small amounts of electron shuttles could sustain flocking, making presence of synthesis and excretion genes in the community expected. We expected the presence and expression of shuttle synthesis and excretion genes in the community. Many of the MAGs expressed flavin synthesis gene *ribBA/X* combined with *yeeO* or *bfe* for export (27, 28, 31). Also phenazine gradients seem plausible, since many bacteria expressed *phzE* and *ipdG*.^15^, ^25^, ^29^ The sediment origin, a eutrophic pond with plants, inhabited by animals, suggests that humic substances, a naturally occurring quinone group-containing electron shuttle, was present (15, 18, 25, 51, 52).

We identified 22 candidate flockers, 21.4% of all MAGs, and of these, 42.1% was likely actively flocking. This amount of flockers may potentially be higher than in fully natural communities due to the reduced community complexity in our cable bacteria enrichment (6). Candidate flockers were classified into 9 phyla and 18 different genera. This taxonomic diversity fits with the previously observed morphological diversity (12, 14). Many of these taxa have been observed in freshwater (8–10, 53) and marine (7, 10, 11) sediment enrichments of cable bacteria.

From the many different interesting taxa with regard to EET and shuttle synthesis, we discuss the top 4 phyla of interest with regard to EET capabilities and correlation with our simplified cable bacteria enrichment. Other taxa (divided per phylum) can be found in the supplementary information (Note S1).

### Acidobacteriota

Two *Acidobacteriota*, a *Holophaga* and a *Geothrix,* were identified as motile. The *Holophaga* expressed homologs of the *extEFG* pcc from the model organism *Geobacter*, the omc *mtrF* of *Shewanella*, and to inner conduits of *Geobacter* and *Shewanella*—two well-described Fe-respiring bacteria (Table S1) (21). *ExtEFG* was significantly more expressed (*P*-values ranged from 3.83E^−08^ to 0.00276) during high cable bacterial abundance (days 26 and 33), suggesting the importance of this pcc for cable bacteria-*Holophaga* interaction. It also transcribed the highest amount of outer membrane EET genes of all MAGs, especially during high cable bacteria abundance (days 26, 33), which suggests that I11_*Holophaga* associates via EET with cable bacteria. The *Geothrix* showed the same potential for EET but we detected expression of only *mtrF* and *fccA*, a conduit. *Holophaga* and *Geothrix* have been implicated in EET before and seen in bioanode communities (54, 55). The *Holophaga* and *Geothrix* may well perform EET as flockers. This may be an interesting synergy where they consume released ferrous iron, stimulated by cable bacteria activity (3, 56, 57), and then donate the gained electrons to cable bacteria, previously also suggested by Otte et al. (10).

### Campylobacterota

One MAG was identified as *Sulfurimonas* sp. (*Campylobacterota*). Colleagues observed that chemoautotrophic sulfur-oxidizing *Epsilonproteobacteria* correlated with and were active in absence of oxygen around both marine and freshwater cable bacteria (7, 8, 10, 11). Direct association with cable bacteria has thus far not been found. The *Sulfurimonas* had pcc, shuttle synthesis, and conduit genes. No pcc was complete, but with genes for *pioA, mtoA, dmsA,* and *dmsE*, it may be using an undetected porin for transmembrane electron transport. Based on 16S rRNA amplicons, the *Sulfurimonas* genus correlated positively with the cable bacteria in the natural lake sediment, suggesting an association. A recently discovered *Sulfurimonas* was shown to oxidize sulfide by transferring electrons to manganese(IV) oxide particles, which requires a form of EET due to the solid nature of this electron acceptor (58). The incomplete pccs of the *Sulfurimonas* leave its potential for IET (interspecies electron transfer) up for question. We found only one MAG in our data sets classified as *Campylobacterota*. The *de novo*-assembled EET genes classified as *Campylobacterota* were thus assumed to belong to the *Sulfurimonas*. The EET genes were mostly transcribed when cable bacteria were highly abundant, suggesting that it was interacting with cable bacteria or used EET during changes brought on by cable bacterial activity.

### Desulfobacterota

Five motile *Desulfobacterota* expressed inner conduits, four of those expressed omcs, and only three (I8, N25, N26) also had genes for two to four pccs, of which two to three were expressed, making these potential flockers (Table S1). These three were *Pseudopelobacteraceae*, the family that *Geobacter* belongs to and which thus contains well-studied EET performers (Table S3) (59–62). I8 significantly (*P*_adj_ = 0.00499) more expressed *omcZ* on days 26 and/or 33 when relative abundance of cable bacteria was high and was the second highest outer membrane EET gene-transcribing MAG (Fig. 3). Thus, it is likely intensively using these proteins during high cable bacterial abundance. As described above, cable bacteria have a complex interaction with the iron cycle, providing several different ways that they could stimulate the activity of an iron oxidizing or reducing microorganism (10). Cable bacteria activity acidifies the sediment, releasing Fe(II) from sources such as FeS (3, 56, 57). Fe(II) oxidation could be coupled to the reduction of cable bacteria cells, resulting in the production of Fe(III) for conventional Fe(III) reduction. Alternatively, one could imagine that iron-cycling microorganisms are not involved in iron cycling at all, but rather couple organic matter oxidation to cable bacteria reduction via EET.

Additionally, cable bacteria have also been shown to indirectly stimulate sulfate-reducing bacteria in freshwater sediments (8, 9). That indirect relationship could potentially be visible in the correlation analyses as positive correlations with the cable bacteria. Two *Desulfobacterota* genera were found to positively correlate with cable bacteria in the enrichment and two in the natural sediment; this is consistent with previously shown correlations (8, 9). However, such correlations do not exclude *Desulfobacterota* from having EET genes nor from being a potential flocking bacterium. Sulfate reduction does not necessarily exclude the potential for IET as some species can use organics, fumarate, antraquinone-2,6-disulfonate (AQDS; two electron shuttles), iron, electrodes, and potentially the cable bacteria as alternative electron acceptors (63, 64).

I12_*Desulfobulbus* and N86_*Humidesulfovibrio* were likely sulfate reducers that correlated with cable bacteria (16S rRNA) and that showed up in the top 5 MAGs that transcribed outer membrane EET genes or shuttle synthesis genes (Fig. 1 to 3). Neither contained potential for a pcc, but N86 expressed an omc (*therJR_2595*) and *dmsA*. Both expressed synthesis genes for flavins and phenazines. The *Humidesulfovibrio* significantly more expressed (*P*_adj_ = 0.0029–0.0353) three *dmsA* and two *ipdG* genes (*P*_adj_ = 0.0082–0.0084) during high cable bacterial abundance (days 26, 33), suggesting that these were used for an important process. It may be able to extracellularly reduce dimethyl sulfoxide (DMSO), like *Shewanella* (65). This is, however, unlikely to occur in the suboxic zone. We suggest that *Humidesulfovibrio* may help produce shuttles but not perform IET as we expect of the flockers (12). Our data strengthen earlier observations where *Desulfobacterota* groups could be interacting with cable bacteria in the anoxic zone (7–9, 11). *De novo*-assembled EET and shuttle synthesis gene transcripts during high cable bacterial abundance were classified as *Desulfovibrionales* and *Desulfobacterales*, which may be used to support IET. Unfortunately, these were not mapped to any MAGs. The *Desulfovibrionales* and *Desulfobacterales* orders have been associated with freshwater cable bacteria before, where they were linked based on sulfate reduction activity (8, 9). Thus, there might be direct cooperation between cable cells and these candidate flockers in the suboxic zone where there is very little sulfide available (2, 66). Sulfate is limited in freshwater environments, yet with the regeneration from cable bacterial sulfide oxidation, many sulfate reducers will be found here and competition for sulfate likely arises (8, 9). Alternatively, these *Desulfobacterota* may not be reducing sulfate at all, but rather directly using the cable bacteria as an electron acceptor. These candidate flockers may use IET as a way to avoid competition with other sulfate reducers (63, 64).

### *Firmicutes* (*Bacillota*)

A wide range of *Firmicutes* have been observed in bio-electrochemical systems, observed individually as electroactive, and studied as model organisms (20, 47, 55, 67, 68). *Firmicutes* were also previously seen enriched in freshwater cable bacteria sediments (8, 53). We observed that they are enriched in our sediments, and many were even correlating with cable bacteria growth in the simplified enrichment and the natural sediment, confirming that the previously found enrichments of *Firmicutes* are likely based on a form of interaction. Most of the amplicon-based positively correlating taxa belonged to the *Clostridia*. Among the eight *Clostridia* MAGs, most EET genes were transcribed on day 3, during low cable bacterial abundance. The genes belonged almost exclusively to the Gram-positive pcc (*dmkA*, *eetB*, *ndh3*) or shuttle synthesis (20). N273_*Clostridia* expressed its pcc completely and N89_*Fusibacter* expressed *dmkA* and *ndh3*. These and N550_*Sporobacter* also transcribed flavin and phenazine synthesis genes. *Fusibacter* belonged to the top 5 MAGs that expressed the most shuttle synthesis genes, which it did mostly during high cable bacterial abundance. N89_*Fusibacter* likely used flavins as intermediaries for IET as genes for flavin synthesis were expressed. It may fulfill an important role by sustaining electron shuttle concentrations. Based on the presence of a pcc, we indicate *Sporobacter* (N550) and *Fusibacter* (N89) and four *Firmicutes* of the *Clostridia* or *Bacillus* classes (N1417, N20, N273, N1017) as candidates flockers.

### *Pseudomonadota*; *Gammaproteobacteria*

We identified 28 *Gammaproteobacteria* of which 18 could perform chemoautotrophic sulfur oxidation, based on their genome (12). Previously, chemoautotrophic sulfur-oxidizing *Gammaproteobacteria* have been observed correlating with and being active in absence of oxygen around marine and freshwater cable bacteria (7, 8, 10, 11), but a direct association has not yet been shown. The limited access to high-quality electron acceptors (oxygen and nitrate/nitrite) where their donors are means that these bacteria needed an alternative electron acceptor (13). As it is expected that sulfide concentrations are low around cable bacteria, these bacteria would actively have to move toward reduced sulfur sources, or may take advantage of the increased sulfate reduction rates (9). Most of the *Gammaproteobacteria* MAGs had genes for shuttle synthesis and some expressed them, of which N310_*Pseudomonas* was the third most shuttle synthesis gene transcriber, suggesting that it was actively producing riboflavin and phenazine, especially on day 33 (Fig. 3; Table S1). Four MAGs had a complete homolog of *E. faecalis’* pcc (I18_*Azonexus*, I9_*Azonexus*, N1098_*Azonexus*, N1784_*Gammaproteobacteria*). Two MAGs contained *mtoBAD* genes like *S. lithotrophicus* (I20_*Azonexus,* I31_*Rhodoferax*). The *Sideroxydans* MAG (I37) did not contain this pcc; it only contained (and expressed) *mtoD* and was thus not included as a candidate flocker. MAG I27_*Rubrivivax* expressed *dmsA, mtrBA,* and *mtrF* which may form a functional hybrid complex where *mtrF* would replace *mtrC* as omc (50). The reduction window of *mtrC* (−500 + 100 mV) is wider than *mtrF’s* (−400 + 50 mV) (19), thus dependent on which shuttles were used, reduction of the shuttles could occur (69). Another potential hybrid complex was identified in N2411, a motile *Nitrosomonas* with *mtoA, therJR_2595,* and *dmsF*, where *dmsF* would function as the integral OM protein, anchoring *mtoA* from the inside, with *therJR_2595* as omc. Under anoxic conditions, *Nitrosomonas europaea* and *Nitrosomonas eutropha* use organics such as acetate, lactate, or pyruvate coupled to nitrite as electron acceptor (70). We suppose that N2411_*Nitrosomonas* can use these organics, too, but coupled to reducing cable bacteria cells, as there is no nitrite available in the suboxic zone (71). We suppose that these hybrid pccs could be functional and include *Nitrosomonas* and *Rubrivivax* as possible flockers. The potential flockers are four *Azonexus* species (I18, I9, N1098, I20), *Nitrosomonas* (N2411), *Rhodoferax* (I31), and *Rubrivivax* (I27). Three of these MAGs (I20, I27, I31) expressed their pcc genes and most transcribed inner conduits. The *Rubrivivax* did not contain enough flagellar assembly genes to assume flagellar motility (23/55); however, this MAG lacked ~20% of its genome and the type strain (*Rhodocyclus gelatinosus*) was described to be motile (72). Therefore, we assumed that it was capable of motility. The genera we found as potential flockers have members which have been verified as EET performers: *Sideroxydans, Azonexus,* and *Rhodoferax* (73–75).

### Conclusion and ecological impact

We assumed that flockers would correlate with cable bacteria, due to their metabolic interaction (14). Our data reinforce this, but also showed that exceptions could exist. In addition, there was a large volume of EET gene transcripts found in the metatranscriptomes, but not mapped to any MAG, suggesting that the diversity of potential flockers is likely larger than the 22 we propose here. The large amount of genes and the expression thereof, combined with the low concentration required for electron shuttles, support the hypothesis that flockers use shuttles as intermediates to transport electrons to cable bacteria (12, 13). Based on taxonomy, the flockers are an extraordinary metabolically flexible group ranging from organotrophs to iron (metal) and sulfur users, but electron donor origins need experimental confirmation. The overlap in metabolic functions of genera that positively correlated with cable bacteria between our sediment enrichment and the natural lake sediment shows that the simplified community in the enrichment still represents natural processes, albeit in potentially higher abundances or lower diversity. The discovery of a large group of likely EET-performing bacteria that could associate with cable bacteria extends the influence of oxygen reduction deep into anoxic sediments. While cable bacteria are only known to directly oxidize sulfide, a direct electric association with other microorganisms widens the range of possible electron donors that could be oxidized in anoxic cable bacteria-containing sediment to include metals and organic compounds. This means that the impact of cable bacteria on carbon and metal cycling could be much greater than previously thought.

## Data Availability

16S rRNA amplicon sequences from all three time series experiments are available at NCBI in the Sequence Read Archive (SRA) and can be found under accession number PRJNA837365 and PRJNA1058976. Metagenomic data is available at the NCBI database under accession number PRJNA730231 (12) and from ENA under bio project number PRJEB52550 (34). Metatranscriptomic data are available from the NCBI SRA-database under accession number PRJNA1057540.

## SUPPLEMENTAL MATERIAL

Figure S1 msystems.01259-24-s0001.docx Setup during microsensor profiling.

Figure S2 msystems.01259-24-s0002.docx Heatmap of time series containing *Ca*. Electronema aureum GS enriched sediment showing the genera that correlated positively with *Ca*. Electronema aureum GS over time.

Figure S3 msystems.01259-24-s0003.docx Heatmap of time series containing *Ca*. Electronema aureum GS enriched sediment showing the genera that correlated positively with *Ca*. Electronema aureum GS over time.

Supplemental text msystems.01259-24-s0004.docx Note S1.

Table S1 msystems.01259-24-s0005.docx Overview of all extracellular electron transfer, shuttle synthesis, and shuttle excretion genes and transcripts thereof that were mapped to the MAGs.

Table S2 msystems.01259-24-s0006.docx All MAGs with their expressed genes per MAG, number of significantly expressed genes during high relative abundance of cable bacteria, and percentage of significantly expressed genes of the expressed genome.

Table S3 msystems.01259-24-s0007.docx Overview of all MAGs and their full taxonomic classification with GTDB-Tk.

ASM does not own the copyrights to Supplemental Material that may be linked to, or accessed through, an article. The authors have granted ASM a non-exclusive, world-wide license to publish the Supplemental Material files. Please contact the corresponding author directly for reuse.
