# Supplementary figures and images for: Extracellular electron transfer genes expressed by candidate flocking bacteria in cable bacteria sediment
Source: mSystems. 2024 Dec 19;10(1):e01259-24. doi: 10.1128/msystems.01259-24 (PMC11748539; doi:10.1128/msystems.01259-24)

**Supplementary information**

**
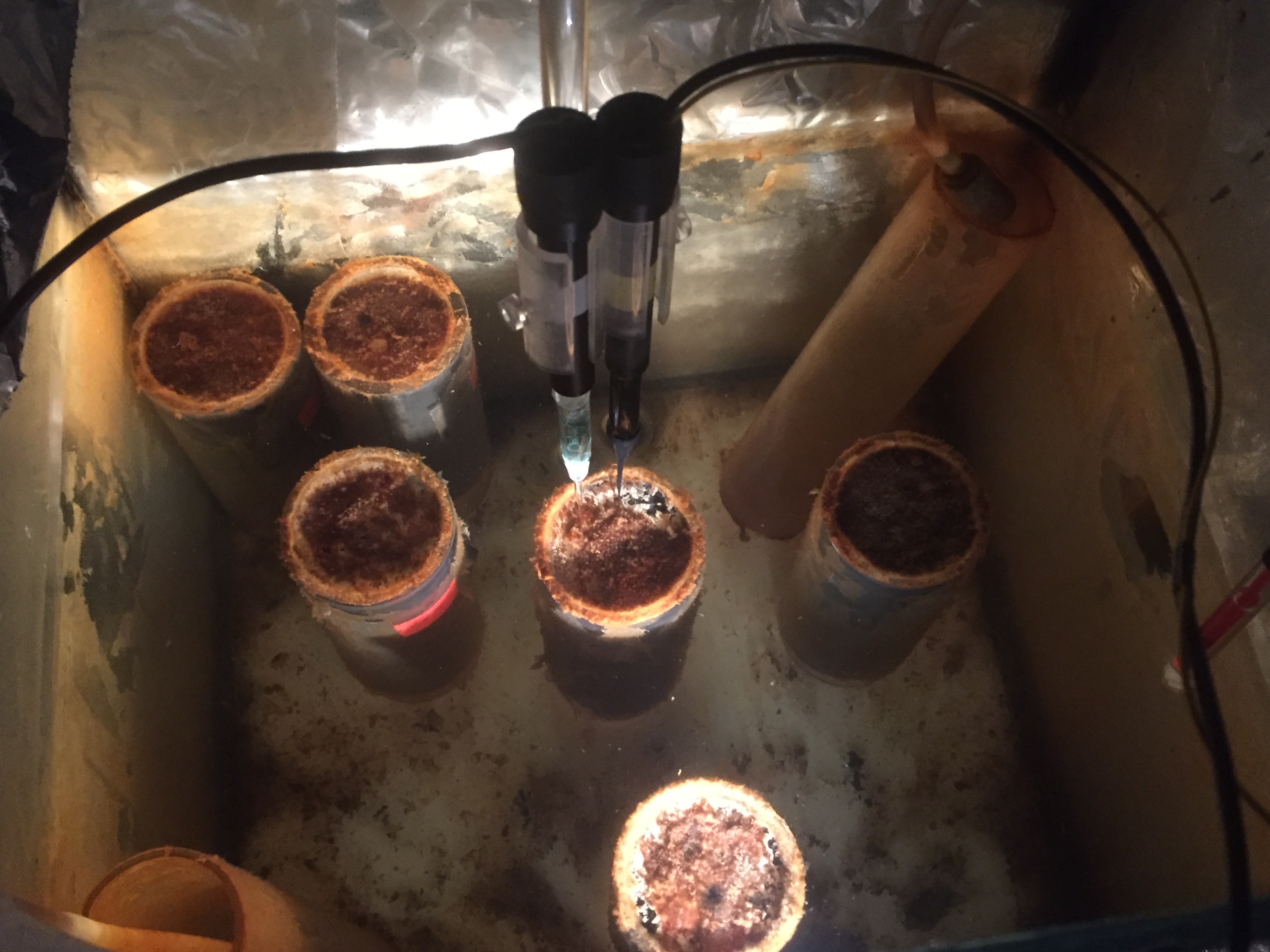

Figure S1 –** Photograph of the set-up during microsensor profiling.

Supplement: Figure S1 — Setup during microsensor profiling. [file msystems.01259-24-s0001.docx]
