# Supplementary material for: Extracellular electron transfer genes expressed by candidate flocking bacteria in cable bacteria sediment
Source: mSystems. 2024 Dec 19;10(1):e01259-24. doi: 10.1128/msystems.01259-24 (PMC11748539; doi:10.1128/msystems.01259-24)
Supplement: Figure S2 — Heatmap of time series containing Ca. Electronema aureum GS enriched sediment showing the genera that correlated positively with Ca. Electronema aureum GS over time. [file msystems.01259-24-s0002.docx]

**Supplementary information**

**
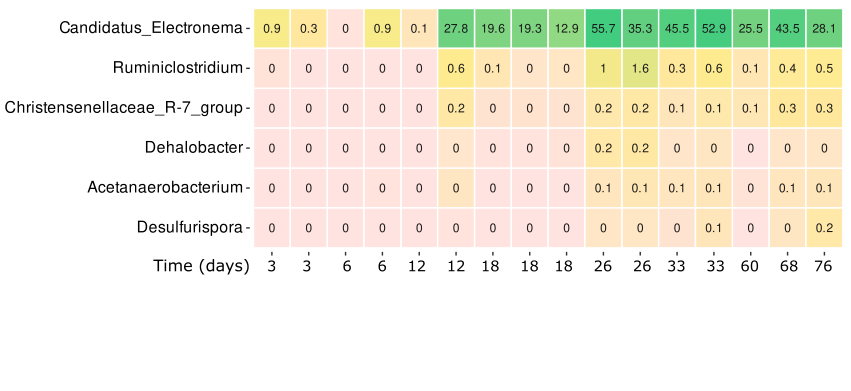
**

**Figure S2 –** Heatmap of time series containing *Ca*. Electronema aureum GS enriched sediment showing the genera that correlated positively with *Ca*. Electronema aureum GS over time (2-76 days, TS1). Numbers on heatmap show percentage abundances.
