# Supplementary material for: Extracellular electron transfer genes expressed by candidate flocking bacteria in cable bacteria sediment
Source: mSystems. 2024 Dec 19;10(1):e01259-24. doi: 10.1128/msystems.01259-24 (PMC11748539; doi:10.1128/msystems.01259-24)
Supplement: Supplemental text — Note S1. [file msystems.01259-24-s0004.docx]

**Supplementary information**

**Supplementary Note 1**

***Actinomycetota.*** We classified two MAGs as *Actinomycetota*, of which one showed potential for EET. It was not classified further, had potential for *Thermincola sp.* pcc-genes such as *cwcA*, *therJR_1117*, *therJR_2595*, transcribed *extEFG* and *mtrF*. A *Rhodococcus* species contained some pcc-related genes, but not enough to form a pcc, it was one of the genera that correlated positively with cable bacteria 16S rRNA. Neither had flagellar motility, excluding them as flockers, but not from associating with cable bacteria.
EET and shuttle synthesis genes were found in the *Coriobacteriia* class within the *Actinomycetota* and were mostly transcribed when cable bacteria where in low abundance, suggesting that a relationship with cable bacteria may have only been feasible in the early stages of their development due to the cable-generated geochemical changes^1,2^.

***Armatimonadota.*** One of two motile *Armatimonadota* MAGs expressed *cyc2* and *mtoA*, but these were not more significantly increased in the presence of cable bacteria. It also encoded *therJR_1117*, *therJR_2595*, inner conduits, and genes for flavin and phenazine synthesis. As *cyc2* was predicted to be a transmembrane beta-barrel protein in *S. lithotrophicus*, this may well function as a stand-alone pcc^3^. Thus this *Armatimonadota* MAG N678 is a candidate flocker.

***Bacteroidota.*** Of 16 *Bacteroidota* MAGs, only I43 contained potential for incomplete pccs (*omabB*, *omabC*, *extG*), while others expressed an omc (*mtrC*, *omcC*, *therJR_2595*). I43 was not further classified than *Bacteroidetes* and with ~17% genome-completeness, the missing EET genes and unclear motility may be explained. The motile N1148_*Bacteroidota* expressed *mtrC*, but also showed potential for other omcs (*pioA, mtoA*) and four inner conduits of which it expressed *macA*. *Bacteroidota* have been implicated with EET before: *Dysgonomonas oryzarvi*, was directly isolated from a microbial fuel cell where it performed EET and *Bacteroidota* have been identified in connection with EET-genes^4-6^. While none of the discussed bacteria show convincing potential for EET, the building blocks appear present and it may well be that the mechanism of I43 was not part of the binned ~17%. We suggest that I43 could potentially be a flocking bacterium.

***Myxococcota.*** Members of the *Myxococcota* were indicated previously to correlate positively with marine cable bacteria^7^. An *Anaeromyxobacter*, a genus in the *Myxococcota*, has been experimentally demonstrated in 2009 to be able to perform EET with radionuclides, and other members of the *Myxococcota* phylum were discovered with EET-potential^4,8^. The motile N75 could perform EET with its partially expressed *omabcB* pathway, making it a candidate flocker.

***Spirochaetota.*** Amongst the four *Spirochaetota*, most could synthesize shuttles, but only one had pcc-genes. N701, a *Treponematales* may be a flocker. This clade was recently connected to genetic EET-potential^4^.

***Sumerlaeota.*** The only *Sumerlaeota* expressed 7 shuttle synthesis-genes, two pcc genes: *pioA* and *omcB*, with genes for *therJR_2595*, *mtrA* and *dmsE*, and inner conduits. No way for electrons to cross into the inner membrane was observed. Pcc-proteins are located in the outer membrane, suggesting a possible alternative way for electrons to cross the inner membrane/periplasm.

***Verrucomicrobiota.*** The *Verrucomicrobiota* showed genes for electron shuttle-synthesis. Their EET-potential was limited to some conduit genes. However, most had *therJR_2595* from the Gram-positive *T. potens JR* and *dmkA* (*E. faecalis*, Gram positive) was identified in two, while the phylum is described to be Gram negative^9,10^. *MtrA* was the only Gram-negative pcc-gene.

***Others.*** A few MAGs were classified with very little potential for EET, but may supply electron shuttles: *Alphaproteobacteria,* *Hydrogenedentota*, *Planctomycetota*, Archaea. A *Patescibacteria* MAG had the highest fraction of its genome significantly more expressed during high cable abundance compared to low cable bacteria abundance (N2834, 41.7%), but did not contain EET-genes or flagella. It may be acclimating to the geochemical changes from cable bacteria activity^1,2,11,12^. A subset of *Alphaproteobacteria* may interact with cable bacteria as iron-metabolizers as previously suggested and were seen to increase with cable bacterial growth^13-15^. However, no pccs were found with exception of a singular genes for *pioC, dmkA*, *dmsE,* 8 *ndh3* genes, and inner conduit genes. *Alphaproteobacteria* were also identified in microbial fuel cell communities^5,16^. Their clear presence may imply a support-role; synthesizing shuttles as at least 13 *Alphaproteobacteria* have the genes for flavins or phenazines, some may also synthesize secondary substrates or perform degradation.

**References accompanying Supplementary Note 1**

1. Pfeffer C, et al. 2012. “Filamentous bacteria transport electrons over centimetre distances,” *Nature*, 491, pp. 218–221. https://doi.org/10.1038/nature11586
2. Risgaard-Petersen N, et al. 2012. “Sulfur, iron-, and calcium cycling associated with natural electric currents running through marine sediment,” *GCA*, 92, pp. 1–13. https://doi.org/10.1016/j.gca.2012.05.036
3. Paquete CM, et al. 2022. Molecular Mechanisms of Microbial Extracellular Electron Transfer. *Frontiers in Bioscience-Landmark*, 27(6), 1-16. https://doi.org/10.31083/j.fbl2706174
4. Olmsted CN, et al. 2022. Environmental predictors of electroactive bacterioplankton in small boreal lakes. *Environ. Microbio* 25(3), p 705-720. https://doi.org/10.1111/1462-2920.16314.
5. Arbour TJ, et al. 2020. Diverse Microorganisms in Sediment and Groundwater Are Implicated in Extracellular Redox Processes Based on Genomic Analysis of Bioanode Communities. *Front. Microbiol* 11, 1694. https://doi.org/10.3389/fmicb.2020.01694
6. Kodama Y, et al. 2021. *Dysgonomonas oryzarvi sp. nov.,* isolated from a microbial fuel cell. *Int. J. Syst. Evol. Microbiol*., 62, 3055-3059. https://doi.org/10.1099/ijs.0.039040-0
7. Liau P, et al. 2022. Microbial succession in a marine sediment: Inferring interspecific microbial interactions with marine cable bacteria. *Envir. Microb*. https://doi.org/10.1111/1462-2920.16230
8. Marshall MJ, et al. 2009. Electron donor-dependent radionuclide reduction and nanoparticle formation by Anaeromyxobacter dehalogenans strain 2CP-C. *Environ Microbiol*. 11(2):534-43. doi: 10.1111/j.1462-2920.2008.01795.x. PMID: 19196283.
9. Hederstedt L, et al. 2020. Two Routes for Extracellular Electron Transfer in *Enterococcus faecalis*. *J. Bacteriol*, 202(7). https://doi.org/10.1128/JB.00725-19
10. Carlson HK, et al. 2012. Surface multiheme c-type cytochromes from *Thermincola potens* and implications for respiratory metal reduction by Gram-positive bacteria. *PNAS*, 109(5), 1702-1707. Doi:10.1073/pnas.1112905109
11. Lustermans JJM, et al. 2023. Persistent flocks of diverse motile bacteria in long-term incubations of electron-conducting cable bacteria, *Candidatus* Electronema aureum. *Front Microbiol*, 14. https://doi.org/10.3389/fmicb.2023.1008293.
12. Rao AMF, et al. 2015. The impact of electrogenic sulfide oxidation on elemental cycling and solute fluxes in coastal sediment. *GCA*, 172, 265–286. https://doi.org/10.1016/j.gca.2015.09.014
13. Sachs C, et al. 2022. Tracing long-distance electron transfer and cable bacteria in freshwater sediments by agar pillar gradient columns. *FEMS Microbiology Ecology* 98, fiac042. https://doi.org/10.1093/femsec/fiac042.
14. Liu F, et al. 2021. “Cable bacteria extend the impacts of elevated dissolved oxygen into anoxic sediments,” *ISMEJ*, 15, pp. 1551–1563. https://doi.org/10.1038/s41396-020-00869-8.
15. Otte JM, et al. 2018. “The distribution of active iron-cycling bacteria in marine and freshwater sediments is decoupled from geochemical gradients,” *Environ Microbiol*, 20(7), pp. 2483–2499. https://doi.org/10.1111/1462-2920.14260.
16. Shi Z, et al. 2021. Small boreholes embedded in the sediment layers make big difference in performance of sediment microbial fuel cells: Bioelectricity generation and microbial community. *Int J Hydrog Energy*, 46(58), 30124-30134. https://doi.org/10.1016/j.ijhydene.2021.06.155
