# Supplementary material for: Extracellular electron transfer genes expressed by candidate flocking bacteria in cable bacteria sediment
Source: mSystems. 2024 Dec 19;10(1):e01259-24. doi: 10.1128/msystems.01259-24 (PMC11748539; doi:10.1128/msystems.01259-24)
Supplement: Table S1 — Overview of all extracellular electron transfer, shuttle synthesis, and shuttle excretion genes and transcripts thereof that were mapped to the MAGs. [file msystems.01259-24-s0005.docx]

**Supplementary information**

**Table S1 –** Overview of all extracellular electron transfer-, shuttle synthesis and shuttle excretion genes* (yellow) and transcripts (green) thereof that were mapped to the MAGs (metagenome-assembled genomes). With their classified phylum and genus, completion, percentage of significantly expressed genome during high cable bacteria abundance (days 26, 33) and count of flagellar genes as per the KEGG database. Numbers represent identified gene-copies.

*Genes used for screening and their origin: *Acidithiobacillus sp.* (OmcS), *A. ferridurans* (Cyc2), *Geobacter sulfurreducens* (OmcS, OmabcB, OmabcC, ExtBCD, ExtEFG, OmcZ, MacA, ImcH, CbcL), *G. metallireducens* (MacA, CbcL), *G. sp.* (PpcABCDE, 2x MacA), *Shewanella oneidensis* MR-1 (MtrABCDEF, OmcA, Bfe, DmsAEF, RibBA), *S. oneidensis* (GspG, FccA), *Aeromonas hydrophila* (OmcA, RibE1,2), *Rhodopseudomonas palustris* TIE-1 (PioABC), *Pseudomonas aeruginosa* (IpdG, PhzABDEF, MexGHI, OpmD), *Pseudomonas fluorescens* (PhzG), *Sideroxydans lithotrophicus* ES-1 (MtoABD, CymA, ImoA), *S. lithotropicus* (ImoA), *Escherichia coli* (YeeO, 2x RibBA, RibD, MenC), *Komagataeibacter europaeus* (PhzF), *Bradyrhizobium japonicum* (NapC), *Acinetobacter baumannii* (RibBX), *Methanococcus vannielii* (Flab3), *Methanothermococcus thermolithotropicus* (FlaB3), *Methanospirillum hungatei* (FlaB3), *Desulfuromonas sp.* (ImcH), *Methanosarcinales sp.* (ImcH), *Thermincola ferriacetica* (ImdcA, PdcA, CwcA), *T. potens* JR (TherJR_0333, TherJR_1117, TherJR_1122, TherJR_2595), *Enterococcus faecalis* (EetB, DmkA, MenBE, Ndh3).
