# Supplementary material for: Extracellular electron transfer genes expressed by candidate flocking bacteria in cable bacteria sediment
Source: mSystems. 2024 Dec 19;10(1):e01259-24. doi: 10.1128/msystems.01259-24 (PMC11748539; doi:10.1128/msystems.01259-24)
Supplement: Table S2 — All MAGs with their expressed genes per MAG, number of significantly expressed genes during high relative abundance of cable bacteria, and percentage of significantly expressed genes of the expressed genome. [file msystems.01259-24-s0006.docx]

**Supplementary information**

**Table S2 –** All MAGs with their expressed genes per MAG (BBmap), number of significantly expressed genes (DESeq2) during high relative abundance of cable bacteria (days 26,33) compared to low relative abundance (day 3) and percentage of significantly expressed genes of the expressed genome.
