# Supplementary material for: Extracellular electron transfer genes expressed by candidate flocking bacteria in cable bacteria sediment
Source: mSystems. 2024 Dec 19;10(1):e01259-24. doi: 10.1128/msystems.01259-24 (PMC11748539; doi:10.1128/msystems.01259-24)
Supplement: Table S3 — Overview of all MAGs and their full taxonomic classification with GTDB-Tk. [file msystems.01259-24-s0007.docx]

**Supplementary information**

**Table S3 –** Overview of all metagenome assembled genome bins (MAGs) and their full taxonomic classification with GTDB-Tk. MAGs with I numbers originate from PRJNA730231 (NCBI; Bjerg et al, 2023) and N numbers from PRJEB52550 (ENA; Sereika et al, 2022).

| Genome_name | Genome_bin_name | Taxonomic classification (GTDB-Tk) |
| --- | --- | --- |
| N1390 | ENR-C_bin.1390 | d__Archaea;p__Halobacteriota;c__Methanosarcinia;o__Methanosarcinales;f__Methanosarcinaceae;g__Methanosarcina;s__ |
| N1827 | ENR-C_bin.1827 | d__Archaea;p__Thermoplasmatota;c__Thermoplasmata;o__Methanomassiliicoccales;f__UBA472;g__;s__ |
| I10 | GS.30 | d__Bacteria;p__Acidobacteriota;c__Holophagae;o__Holophagales;f__Holophagaceae;g__Geothrix;s__ |
| I11 | GS.11 | d__Bacteria;p__Acidobacteriota;c__Holophagae;o__Holophagales;f__Holophagaceae;g__Holophaga;s__ |
| I1 | GS.1 | d__Bacteria;p__Actinobacteriota;c__Actinomycetia;o__Mycobacteriales;f__Mycobacteriaceae;g__Rhodococcus;s__Rhodococcus qingshengii |
| N2538 | ENR-C_bin.2538 | d__Bacteria;p__Actinobacteriota;c__Coriobacteriia;o__OPB41;f__PHET01;g__;s__ |
| N1744 | ENR-C_bin.1744 | d__Bacteria;p__Armatimonadota;c__HRBIN16;o__HRBIN16;f__HRBIN16;g__;s__ |
| N678 | ENR-C_bin.678 | d__Bacteria;p__Armatimonadota;c__UBA5829;o__UBA5829;f__UBA5829;g__;s__ |
| N100 | ENR-C_bin.100 | d__Bacteria;p__Bacteroidota;c__Bacteroidia;o__Bacteroidales;f__Paludibacteraceae;g__Paludibacter;s__ |
| N404 | ENR-C_bin.404 | d__Bacteria;p__Bacteroidota;c__Bacteroidia;o__Bacteroidales;f__Prolixibacteraceae;g__GWE2-42-39;s__ |
| N23 | ENR-C_bin.23 | d__Bacteria;p__Bacteroidota;c__Bacteroidia;o__Bacteroidales;f__Prolixibacteraceae;g__JAAFHB01;s__ |
| N113 | ENR-C_bin.113 | d__Bacteria;p__Bacteroidota;c__Bacteroidia;o__Bacteroidales;f__UBA12481;g__UBA12481;s__ |
| N342 | ENR-C_bin.342 | d__Bacteria;p__Bacteroidota;c__Bacteroidia;o__Bacteroidales;f__UBA1402;g__UBA8389;s__ |
| N194 | ENR-C_bin.194 | d__Bacteria;p__Bacteroidota;c__Bacteroidia;o__Bacteroidales;f__UBA932;g__Bact-08;s__ |
| N19 | ENR-C_bin.19 | d__Bacteria;p__Bacteroidota;c__Bacteroidia;o__Bacteroidales;f__VadinHA17;g__LD21;s__ |
| N151 | ENR-C_bin.151 | d__Bacteria;p__Bacteroidota;c__Bacteroidia;o__Bacteroidales;f__WCHB1-69;g__UBA5429;s__ |
| N1114 | ENR-C_bin.1114 | d__Bacteria;p__Bacteroidota;c__Bacteroidia;o__Cytophagales;f__Cyclobacteriaceae;g__Algoriphagus;s__ |
| N628 | ENR-C_bin.628 | d__Bacteria;p__Bacteroidota;c__Bacteroidia;o__Flavobacteriales;f__Flavobacteriaceae;g__Flavobacterium;s__ |
| N1440 | ENR-C_bin.1440 | d__Bacteria;p__Bacteroidota;c__Bacteroidia;o__Sphingobacteriales;f__Sphingobacteriaceae;g__Pedobacter;s__ |
| N1148 | ENR-C_bin.1148 | d__Bacteria;p__Bacteroidota;c__Kapabacteria;o__Kapabacteriales;f__UBA2268;g__;s__ |
| N587 | ENR-C_bin.587 | d__Bacteria;p__Bacteroidota;c__Kapabacteria;o__Kapabacteriales;f__UBA2268;g__;s__ |
| I28 | GS.28 | d__Bacteria;p__Bacteroidota;c__UBA10030;o__UBA10030;f__UBA8401;g__CAISDA01;s__ |
| I43 | GS.43 | d__Bacteria;p__Bacteroidota;c__UBA10030;o__UBA10030;f__UBA8401;g__CAISDA01;s__ |
| I21 | GS.21 | d__Bacteria;p__Bacteroidota;c__UBA10030;o__UBA10030;f__UBA8401;g__UBA8401;s__ |
| N426 | ENR-C_bin.426 | d__Bacteria;p__Campylobacterota;c__Campylobacteria;o__Campylobacterales;f__Sulfurimonadaceae;g__Sulfurimonas;s__ |
| N87 | ENR-C_bin.87 | d__Bacteria;p__Chloroflexota;c__Anaerolineae;o__Anaerolineales;f__EnvOPS12;g__OLB14;s__ |
| I12 | GS.12 | d__Bacteria;p__Desulfobacterota;c__Desulfobulbia;o__Desulfobulbales;f__Desulfobulbaceae;g__Desulfobulbus;s__ |
| NENR | ENR-cMAG | d__Bacteria;p__Desulfobacterota;c__Desulfobulbia;o__Desulfobulbales;f__Desulfobulbaceae;g__Electronema;s__Electronema sp004284765 |
| N26 | ENR-C_bin.26 | d__Bacteria;p__Desulfobacterota;c__Desulfuromonadia;o__Geobacterales;f__Pseudopelobacteraceae;g__CAIPTY01;s__ |
| N25 | ENR-C_bin.25 | d__Bacteria;p__Desulfobacterota;c__Desulfuromonadia;o__Geobacterales;f__Pseudopelobacteraceae;g__JACRCG01;s__ |
| I8 | GS.8 | d__Bacteria;p__Desulfobacterota;c__Desulfuromonadia;o__Geobacterales;f__Pseudopelobacteraceae;g__JACRCG01;s__ |
| N86 | ENR-C_bin.86 | d__Bacteria;p__Desulfobacterota_I;c__Desulfovibrionia;o__Desulfovibrionales;f__Desulfovibrionaceae;g__Humidesulfovibrio;s__ |
| N326 | ENR-C_bin.326 | d__Bacteria;p__Firmicutes;c__Bacilli;o__Erysipelotrichales;f__Erysipelotrichaceae;g__GWE2-51-13;s__ |
| N1017 | ENR-C_bin.1017 | d__Bacteria;p__Firmicutes;c__Bacilli;o__Erysipelotrichales;f__Erysipelotrichaceae;g__UBA2212;s__ |
| N1073 | ENR-C_bin.1073 | d__Bacteria;p__Firmicutes;c__Bacilli;o__Erysipelotrichales;f__Erysipelotrichaceae;g__UBA2212;s__ |
| N1054 | ENR-C_bin.1054 | d__Bacteria;p__Firmicutes;c__Bacilli;o__Izemoplasmatales;f__UBA5603;g__UBA5603;s__ |
| N2571 | ENR-C_bin.2571 | d__Bacteria;p__Firmicutes;c__Bacilli;o__RF39;f__UBA660;g__;s__ |
| N273 | ENR-C_bin.273 | d__Bacteria;p__Firmicutes_A;c__Clostridia;o__Acetivibrionales;f__DSM-8532;g__;s__ |
| N20 | ENR-C_bin.20 | d__Bacteria;p__Firmicutes_A;c__Clostridia;o__Acetivibrionales;f__DTU013;g__;s__ |
| N540 | ENR-C_bin.540 | d__Bacteria;p__Firmicutes_A;c__Clostridia;o__Christensenellales;f__CAG-138;g__UBA7703;s__ |
| N1417 | ENR-C_bin.1417 | d__Bacteria;p__Firmicutes_A;c__Clostridia;o__Christensenellales;f__QAND01;g__RGIG3178;s__ |
| N152 | ENR-C_bin.152 | d__Bacteria;p__Firmicutes_A;c__Clostridia;o__Lutisporales;f__Lutisporaceae;g__GWB2-37-7;s__ |
| N550 | ENR-C_bin.550 | d__Bacteria;p__Firmicutes_A;c__Clostridia;o__Oscillospirales;f__Oscillospiraceae;g__Sporobacter;s__ |
| N89 | ENR-C_bin.89 | d__Bacteria;p__Firmicutes_A;c__Clostridia;o__Peptostreptococcales;f__Acidaminobacteraceae;g__Fusibacter_C;s__ |
| N2038 | ENR-C_bin.2038 | d__Bacteria;p__Firmicutes_A;c__Clostridia;o__Saccharofermentanales;f__UBA5734;g__UBA8949;s__ |
| N554 | ENR-C_bin.554 | d__Bacteria;p__Hydrogenedentota;c__Hydrogenedentia;o__Hydrogenedentiales;f__WGMK01;g__JAEUWI01;s__ |
| N75 | ENR-C_bin.75 | d__Bacteria;p__Myxococcota;c__UBA9042;o__PHBI01;f__PHBI01;g__;s__ |
| N2676 | ENR-C_bin.2676 | d__Bacteria;p__Patescibacteria;c__ABY1;o__BM507;f__UBA12465;g__VFKX01;s__ |
| N2834 | ENR-C_bin.2834 | d__Bacteria;p__Patescibacteria;c__Paceibacteria;o__UBA9983_A;f__XYD1-FULL-46-19;g__;s__ |
| N796 | ENR-C_bin.796 | d__Bacteria;p__Planctomycetota;c__Planctomycetia;o__Planctomycetales;f__Planctomycetaceae;g__;s__ |
| I35 | GS.35 | d__Bacteria;p__Proteobacteria;c__Alphaproteobacteria;o__Caulobacterales;f__Caulobacteraceae;g__Brevundimonas;s__Brevundimonas sp004296955 |
| N1561 | ENR-C_bin.1561 | d__Bacteria;p__Proteobacteria;c__Alphaproteobacteria;o__Caulobacterales;f__Caulobacteraceae;g__Caulobacter;s__ |
| N559 | ENR-C_bin.559 | d__Bacteria;p__Proteobacteria;c__Alphaproteobacteria;o__Caulobacterales;f__Caulobacteraceae;g__Phenylobacterium;s__ |
| I10 | GS.10 | d__Bacteria;p__Proteobacteria;c__Alphaproteobacteria;o__Caulobacterales;f__Caulobacteraceae;g__Phenylobacterium;s__ |
| I40 | GS.40 | d__Bacteria;p__Proteobacteria;c__Alphaproteobacteria;o__Caulobacterales;f__Caulobacteraceae;g__Phenylobacterium;s__ |
| N1511 | ENR-C_bin.1511 | d__Bacteria;p__Proteobacteria;c__Alphaproteobacteria;o__Caulobacterales;f__Hyphomonadaceae;g__UBA7672;s__ |
| I14 | GS.14 | d__Bacteria;p__Proteobacteria;c__Alphaproteobacteria;o__Ferrovibrionales;f__Ferrovibrionaceae;g__Ferrovibrio;s__Ferrovibrio sp002482765 |
| N1493 | ENR-C_bin.1493 | d__Bacteria;p__Proteobacteria;c__Alphaproteobacteria;o__Micropepsales;f__Micropepsaceae;g__Rhizomicrobium;s__ |
| I7 | GS.7 | d__Bacteria;p__Proteobacteria;c__Alphaproteobacteria;o__Rhodospirillales;f__Magnetospirillaceae;g__Phaeospirillum;s__ |
| N1167 | ENR-C_bin.1167 | d__Bacteria;p__Proteobacteria;c__Alphaproteobacteria;o__Sphingomonadales;f__Sphingomonadaceae;g__Alteraurantiacibacter;s__ |
| I38 | GS.38 | d__Bacteria;p__Proteobacteria;c__Alphaproteobacteria;o__Sphingomonadales;f__Sphingomonadaceae;g__Sphingobium;s__Sphingobium limneticum |
| I6 | GS.6 | d__Bacteria;p__Proteobacteria;c__Alphaproteobacteria;o__Sphingomonadales;f__Sphingomonadaceae;g__Sphingobium;s__Sphingobium psychrophilum |
| N357 | ENR-C_bin.357 | d__Bacteria;p__Proteobacteria;c__Alphaproteobacteria;o__Sphingomonadales;f__Sphingomonadaceae;g__Sphingomonas;s__ |
| I24 | GS.24 | d__Bacteria;p__Proteobacteria;c__Alphaproteobacteria;o__Sphingomonadales;f__Sphingomonadaceae;g__Sphingomonas;s__ |
| N334 | ENR-C_bin.334 | d__Bacteria;p__Proteobacteria;c__Alphaproteobacteria;o__Sphingomonadales;f__Sphingomonadaceae;g__Sphingorhabdus_B;s__Sphingorhabdus_B lacus |
| N386 | ENR-C_bin.386 | d__Bacteria;p__Proteobacteria;c__Alphaproteobacteria;o__Sphingomonadales;f__Sphingomonadaceae;g__Sphingosinicella;s__ |
| I4 | GS.4 | d__Bacteria;p__Proteobacteria;c__Gammaproteobacteria;o__Burkholderiales;f__Burkholderiaceae;g__Acidovorax;s__Acidovorax sp001411535 |
| I2 | GS.2 | d__Bacteria;p__Proteobacteria;c__Gammaproteobacteria;o__Burkholderiales;f__Burkholderiaceae;g__Acidovorax;s__Acidovorax sp001428665 |
| I23 | GS.23 | d__Bacteria;p__Proteobacteria;c__Gammaproteobacteria;o__Burkholderiales;f__Burkholderiaceae;g__Aquabacterium;s__Aquabacterium sp001770775 |
| I3 | GS.3 | d__Bacteria;p__Proteobacteria;c__Gammaproteobacteria;o__Burkholderiales;f__Burkholderiaceae;g__Comamonas;s__Comamonas acidovorans (lit-sulfite ox) |
| I15 | GS.15 | d__Bacteria;p__Proteobacteria;c__Gammaproteobacteria;o__Burkholderiales;f__Burkholderiaceae;g__Hylemonella;s__Hylemonella sp013334205 |
| I26 | GS.26 | d__Bacteria;p__Proteobacteria;c__Gammaproteobacteria;o__Burkholderiales;f__Burkholderiaceae;g__Limnobacter;s__Limnobacter sp000170915 |
| I25 | GS.25 | d__Bacteria;p__Proteobacteria;c__Gammaproteobacteria;o__Burkholderiales;f__Burkholderiaceae;g__Malikia;s__Malikia spinosa |
| N710 | ENR-C_bin.710 | d__Bacteria;p__Proteobacteria;c__Gammaproteobacteria;o__Burkholderiales;f__Burkholderiaceae;g__QZKY01;s__ |
| I31 | GS.31 | d__Bacteria;p__Proteobacteria;c__Gammaproteobacteria;o__Burkholderiales;f__Burkholderiaceae;g__Rhodoferax;s__ |
| I27 | GS.27 | d__Bacteria;p__Proteobacteria;c__Gammaproteobacteria;o__Burkholderiales;f__Burkholderiaceae;g__Rubrivivax;s__ |
| I5 | GS.5 | d__Bacteria;p__Proteobacteria;c__Gammaproteobacteria;o__Burkholderiales;f__Burkholderiaceae;g__UBA2334;s__ |
| N1783 | ENR-C_bin.1783 | d__Bacteria;p__Proteobacteria;c__Gammaproteobacteria;o__Burkholderiales;f__Gallionellaceae;g__Nitrotoga;s__ (lit-sulfite ox) |
| I36 | GS.36 | d__Bacteria;p__Proteobacteria;c__Gammaproteobacteria;o__Burkholderiales;f__Gallionellaceae;g__PALSA-1006;s__ |
| I37 | GS.37 | d__Bacteria;p__Proteobacteria;c__Gammaproteobacteria;o__Burkholderiales;f__Gallionellaceae;g__Sideroxydans;s__ |
| I57 | GS.57 | d__Bacteria;p__Proteobacteria;c__Gammaproteobacteria;o__Burkholderiales;f__Methylophilaceae;g__Methylotenera;s__ |
| I34 | GS.34 | d__Bacteria;p__Proteobacteria;c__Gammaproteobacteria;o__Burkholderiales;f__Methylophilaceae;g__Methylotenera;s__Methylotenera mobilis_B |
| N2411 | ENR-C_bin.2411 | d__Bacteria;p__Proteobacteria;c__Gammaproteobacteria;o__Burkholderiales;f__Nitrosomonadaceae;g__Nitrosomonas;s__Nitrosomonas sp902826115 (lit-S0 ox?) |
| N1098 | ENR-C_bin.1098 | d__Bacteria;p__Proteobacteria;c__Gammaproteobacteria;o__Burkholderiales;f__Rhodocyclaceae;g__Azonexus;s__ |
| I18 | GS.18 | d__Bacteria;p__Proteobacteria;c__Gammaproteobacteria;o__Burkholderiales;f__Rhodocyclaceae;g__Azonexus;s__ |
| I9 | GS.9 | d__Bacteria;p__Proteobacteria;c__Gammaproteobacteria;o__Burkholderiales;f__Rhodocyclaceae;g__Azonexus;s__ |
| I20 | GS.20 | d__Bacteria;p__Proteobacteria;c__Gammaproteobacteria;o__Burkholderiales;f__Rhodocyclaceae;g__Azonexus;s__Azonexus sp008080565 |
| I19 | GS.19 | d__Bacteria;p__Proteobacteria;c__Gammaproteobacteria;o__Burkholderiales;f__Rhodocyclaceae;g__CAJAWW01;s__ |
| I17 | GS.17 | d__Bacteria;p__Proteobacteria;c__Gammaproteobacteria;o__Burkholderiales;f__Rhodocyclaceae;g__Methyloversatilis;s__Methyloversatilis sp016791045 |
| N1784 | ENR-C_bin.1784 | d__Bacteria;p__Proteobacteria;c__Gammaproteobacteria;o__Burkholderiales;f__Rhodocyclaceae;g__UBA2250;s__ |
| N651 | ENR-C_bin.651 | d__Bacteria;p__Proteobacteria;c__Gammaproteobacteria;o__Burkholderiales;f__Thiobacillaceae;g__Thiobacillus;s__ |
| N210 | ENR-C_bin.210 | d__Bacteria;p__Proteobacteria;c__Gammaproteobacteria;o__Enterobacterales;f__Alteromonadaceae;g__Pararheinheimera;s__ |
| I42 | GS.42 | d__Bacteria;p__Proteobacteria;c__Gammaproteobacteria;o__Enterobacterales;f__Alteromonadaceae;g__Pararheinheimera;s__ |
| N310 | ENR-C_bin.310 | d__Bacteria;p__Proteobacteria;c__Gammaproteobacteria;o__Pseudomonadales;f__Pseudomonadaceae;g__Pseudomonas_K;s__ (lit-Pseudomonas) |
| N1563 | ENR-C_bin.1563 | d__Bacteria;p__Spirochaetota;c__Leptospirae;o__Turneriellales;f__Turneriellaceae;g__Turneriella;s__ |
| N701 | ENR-C_bin.701 | d__Bacteria;p__Spirochaetota;c__Spirochaetia;o__Treponematales;f__Treponemataceae;g__Spiro-10;s__ |
| I46 | GS.46 | d__Bacteria;p__Spirochaetota;c__Spirochaetia;o__Treponematales;f__UBA12059;g__SZUA-370;s__ |
| N1443 | ENR-C_bin.1443 | d__Bacteria;p__Spirochaetota;c__UBA4802;o__UBA4802;f__UBA5550;g__WRGT01;s__ |
| N1301 | ENR-C_bin.1301 | d__Bacteria;p__Sumerlaeota;c__Sumerlaeia;o__UBA8349;f__;g__;s__ |
| N348 | ENR-C_bin.348 | d__Bacteria;p__Verrucomicrobiota;c__Kiritimatiellae;o__RFP12;f__UBA1067;g__CAIZQW01;s__ |
| N183 | ENR-C_bin.183 | d__Bacteria;p__Verrucomicrobiota;c__Verrucomicrobiae;o__Pedosphaerales;f__UBA8199;g__;s__ |
| N242 | ENR-C_bin.343 | d__Bacteria;p__Verrucomicrobiota;c__Verrucomicrobiae;o__Verrucomicrobiales;f__V1-33;g__CAIZWU01;s__ |
| N491 | ENR-C_bin.491 | d__Bacteria;p__Verrucomicrobiota;c__Verrucomicrobiae;o__Verrucomicrobiales;f__Verrucomicrobiaceae;g__Prosthecobacter;s__ |
| unused | GS.44 | Unclassified |
| unused | GS.48 | Unclassified |
| unused | GS.51 | Unclassified |
| unused | GS.53 | Unclassified |
| unused | GS.54 | Unclassified |
| unused | GS.60 | Unclassified |
| unused | GS.61 | Unclassified |
| unused | GS.62 | Unclassified |
| unused | GS.63 | Unclassified |
| unused | GS.45 | Unclassified Bacteria |
| unused | GS.47 | Unclassified Bacteria |
| unused | GS.50 | Unclassified Bacteria |
| unused | GS.52 | Unclassified Bacteria |
| unused | GS.55 | Unclassified Bacteria |
| unused | GS.56 | Unclassified Bacteria |
| unused | GS.58 | Unclassified Bacteria |
| unused | GS.59 | Unclassified Bacteria |
| unused | GS.64 | Unclassified Bacteria |
